# Effects of Low Dose Space Radiation Exposures on the Splenic Metabolome

**DOI:** 10.3390/ijms22063070

**Published:** 2021-03-17

**Authors:** Evagelia C. Laiakis, Igor Shuryak, Annabella Deziel, Yi-Wen Wang, Brooke L. Barnette, Yongjia Yu, Robert L. Ullrich, Albert J. Fornace, Mark R. Emmett

**Affiliations:** 1 Lombardi Comprehensive Cancer Center, Department of Oncology, Georgetown University, Washington, DC 20057, USA; ad1565@georgetown.edu (A.D.); yiswe421@gmail.com (Y.-W.W.); af294@georgetown.edu (A.J.F.J.); 2 Department of Biochemistry and Molecular & Cellular Biology, Georgetown University, Washington, DC 20057, USA; 3 Center for Radiological Research, Columbia University, New York, NY 10032, USA; is144@cumc.columbia.edu; 4 Department of Biochemistry and Molecular Biology, University of Texas Medical Branch, Galveston, TX 77555, USA; Brlawson@utmb.edu (B.L.B.); yoyu@utmb.edu (Y.Y.); mremmett@utmb.edu (M.R.E.); 5 Radiation Effects Research Foundation, Hiroshima 732-0815, Japan; ullrich@rerf.or.jp; 6 Department of Radiation Oncology, University of Texas Medical Branch, Galveston, TX 77555, USA

**Keywords:** space radiation, spleen, immune, metabolism, mitochondria

## Abstract

Future space missions will include a return to the Moon and long duration deep space roundtrip missions to Mars. Leaving the protection that Low Earth Orbit provides will unavoidably expose astronauts to higher cumulative doses of space radiation, in addition to other stressors, e.g., microgravity. Immune regulation is known to be impacted by both radiation and spaceflight and it remains to be seen whether prolonged effects that will be encountered in deep space can have an adverse impact on health. In this study, we investigated the effects in the overall metabolism of three different low dose radiation exposures (γ-rays, ^16^O, and ^56^Fe) in spleens from male C57BL/6 mice at 1, 2, and 4 months after exposure. Forty metabolites were identified with significant enrichment in purine metabolism, tricarboxylic acid cycle, fatty acids, acylcarnitines, and amino acids. Early perturbations were more prominent in the γ irradiated samples, while later responses shifted towards more prominent responses in groups with high energy particle irradiations. Regression analysis showed a positive correlation of the abundance of identified fatty acids with time and a negative association with γ-rays, while the degradation pathway of purines was positively associated with time. Taken together, there is a strong suggestion of mitochondrial implication and the possibility of long-term effects on DNA repair and nucleotide pools following radiation exposure.

## 1. Introduction

Long duration and deep space travel are in the immediate National Aeronautics and Space Administration (NASA) plans for space exploration and research. Astronauts in future missions to the Moon, the Lunar Gateway, or to Mars will spend a significant amount of time being exposed to the harsh space environment. The space environment includes prolonged presence in microgravity, which on its own can lead to significant physiological changes [1,2], and exposures to space radiation, such as high energy (HZE) particles from galactic cosmic rays (GCRs) and potential particles from solar events (SPE) [3]. Unlike the International Space Station (ISS) that resides on Low Earth Orbit (LEO), where a certain level of protection from radiation exposure is provided due to Earth’s magnetic field [3], future missions will take place in deep space; therefore, astronauts will receive a substantial cumulative dose on an estimated three year mission. From astronaut health evaluations and animal experiments, both in space and on the ground, it is known that spaceflight and/or space radiation exposure have the potential to lead to significant effects such as cardiovascular and central nervous system effects, muscle atrophy, cataract formation, and, importantly, immune system effects, among others [1,4,5,6,7,8,9,10,11,12,13]. During a long duration mission, such physiological effects may prove detrimental to the completion of the tasks at hand and leave astronauts experiencing long-term health effects, as exposure to space radiation remains a significant risk. Therefore, substantial research efforts have been undertaken to understand the space flight environment and, in particular, the effects of the individual HZE particles and other types of space radiation on cellular and tissue responses. In this study, we investigated long-term effects of HZE and γ irradiations on the mouse spleen with untargeted metabolomic profiling. Studies utilizing this omics approach have already focused on various tissues (e.g., gastrointestinal tissue, cardiovascular, biofluids, T-cells, and even the microbiome) [14,15,16,17,18,19], highlighting early and persistent changes; however, this is the first study to focus on the splenic metabolome.

Changes in immune responses have been well documented in astronauts. Immune system dysregulation manifesting as viral reactivation [20,21] or altered adaptive or innate responses in astronauts and *in vivo* and *in vitro* models and cytokine levels [12,13,15,22,23,24,25,26], including responses to space radiation, can lead to short term or persistent effects. As mentioned above, responses to space flight are complex, with radiation exposure contributing substantially to tissue effects, although the effect is poorly understood, therefore complicating risk assessment. Whether space radiation has an additive or synergistic effect with microgravity remains to be determined, particularly in long duration missions and specifically in the immune system. In future missions, such perturbations can prove detrimental to the health of the astronauts and may leave them with lingering issues for multiple years after their return to Earth. Parts of the immune system include circulating cells (e.g., lymphocytes, macrophages, neutrophils), while other cells reside in the bone marrow (i.e., hematopoietic stem cells), thymus, lymph nodes, and spleen. The spleen in particular consists of the red pulp, the white pulp, and the marginal zone that is the interphase between the two [27]. Important functions of the spleen include the filtering of the blood and the recycling of iron, but also storing T-cells, B-cells, dendritic cells, and macrophages [27]. Given this, the spleen is an important organ in fighting infections from bacteria and viruses [27], and, therefore, perturbations by exogenous genotoxic factors, such as ionizing radiation (IR), may lead to organ dysfunction. Data from low dose rate exposures [28] or high dose acute exposures [29,30,31] have shown significant effects on the spleen, including altered metabolism.

The space radiation environment is complex, as GCRs consist of various HZE particles in addition to protons, mixed together with γ-rays and neutrons. Studies with low dose radiation exposure have shown that IR leads to metabolic reprogramming, limiting the efficiency of T-cell activation [19]. Spaceflight can also lead to increased fatty acid oxidation and glycolysis-related profiles, as seen in mouse spleen samples from STS-135 [11]. Nonetheless, space radiation remains one of the highest risks for astronauts. In order to dissect the potential effects of space radiation on long-term immune responses, *in vivo* irradiations were conducted at the NASA Space Radiation Research Laboratory (NSRL) at Brookhaven National Laboratory (BNL). In this study, C57BL/6 male mice were irradiated with low doses of γ-rays, ^56^Fe (600 MeV/n), or ^16^O (1 GeV/n) with doses that could be accumulated during a long duration mission. Small molecules (<1 kDa) were evaluated with metabolomic profiling from samples collected at 1, 2, and 4 months after exposure. Specific perturbations emerged in select metabolic pathways, with purine metabolism and the tricarboxylic acid (TCA) cycle showing the most robust enrichment and exhibiting differences based on radiation quality. Such persistent alterations in key pathways may have long lasting effects on blood detoxification and immune reserves, leading to diminished responses in fighting infections. These unique findings of longitudinal effects of low dose space radiation on metabolism in the spleen provide significant knowledge in the persistent damage that astronauts may accumulate in a deep space mission with relevant accumulated doses.

## 2. Materials and Methods

### 2.1. Chemicals

All chemicals were of the highest purity and all solvents were LC-MS grade. Internal standards (debrisoquine sulfate and 4-nitrobenzoic acid), in addition to pure chemicals (as seen in Table 1) utilized for tandem mass spectrometry (MS/MS), were ordered from Sigma Aldrich (Sigma Aldrich, St. Louis, MO, USA).

### 2.2. Animals and Irradiations

**Figure 1:**
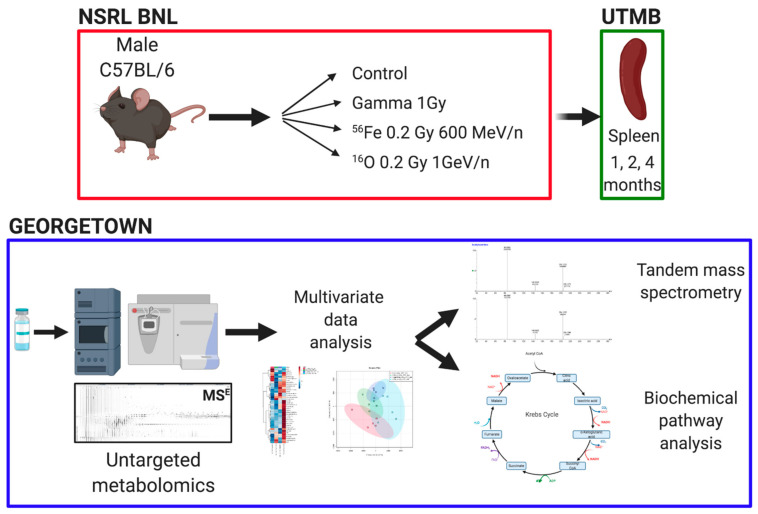
Experimental design. C57BL/6 were exposed to HZE particle irradiations or γ-rays at the NASA Space Radiation Laboratory at Brookhaven National lab. Spleen samples were collected at University of Texas Medical Branch and shipped to Georgetown University, where untargeted metabolomics and data analyses were conducted. Figure was created with BioRender.com (accessed on 25 January 2021).

Male C57BL/6 mice (8–10 weeks old) were ordered from Charles River and shipped directly to Brookhaven National Laboratory (BNL). All studies received University of Texas Medical Branch (UTMB) (protocol #1411064, approved 1 November 2014) and BNL (protocol #480, approved 5 February 2015) Institutional Animal Care and Use Committee (IACUC) approval. Both facilities are AAALAC accredited. Irradiations were performed at the NASA Space Radiation Laboratory (NSRL) as previously described [32] with established dosimetry methods γ irradiations with a ^137^Cs source were also performed at BNL. Mice (*n* = 4–5 per group) were total body irradiated with either 0.2 Gy of ^16^O (1 GeV/n), 0.2 Gy of ^56^Fe (600 MeV/n), 1 Gy ^137^Cs or sham irradiated (controls) and shipped to UTMB. The HZE doses were chosen to reflect total accumulated expected dose from a minimum of a 2-year mission, while the γ dose was chosen as a reference [33]. Timed sacrifice was performed at 1, 2, and 4 months after exposure with weight of the mice recorded at the time of euthanasia, which was performed with CO_2_ asphyxiation under standard humane conditions. Spleen samples were flash frozen and stored at −80 °C until shipment to Georgetown University for metabolomic processing. A schematic of the experimental processing is shown in Figure 1. The radiation scheme described here was designed for a parent study on hepatocellular carcinoma [34,35], with spleen analysis emerging to maximize experimental analysis from a single animal study.

### 2.3. Untargeted Metabolomics and Data Analysis

Metabolite extraction was performed based on previously published protocols [36]. Briefly, a small piece of ~10 mg of tissue was homogenized in methanol:water (*v:v*, 1:1) containing 30 μM debrisoquine sulfate and 4 μM 4-nitrobenzoic acid as internal standards. Samples were centrifuged and the supernatant was transferred to a clean tube. Acetonitrile:water (*v:v*, 1:1) was added to the pellet and the procedure repeated. The two supernatants were combined and vacuum dried with no heat. The dried samples were resuspended in methanol:water (*v:v*, 1:1), filtered through a 0.2 μm sized filter, and transferred to a sample vial. A total of 2 μl of each sample were injected into an Ultra Performance Liquid Chromatography (UPLC) coupled to a time-of-flight mass spectrometer Xevo G2 (Waters Corp., Inc., Milford, MA, USA). The conditions used for analysis are described in Supplementary Table S1. All data were acquired in MS^E^ mode. Quality controls (QC) from pooled samples were injected every 19 samples. Deconvolution was performed with the software Progenesis QI (NonLinear Dynamics, Inc, Newcastle, UK), with peak alignment based on a QC chromatogram chosen by the software, and normalization was performed with the function “normalize to all compounds”. Putative identities were assigned through the databases METLIN MS/MS empirical library [37], Human Metabolome Database (HMDB) [38], and LIPID MAPS [39] with a <10 ppm error.

Multivariate data analysis for positive (ESI^+^) and negative (ESI^−^) data was conducted through MetaboAnalyst 4.0 [40] separately for each of the three time points. Features with >70% missing values were excluded, as were remaining variables with missing values. Data filtering was performed with interquantile range (IQR) and the data were Pareto scaled. One-way analysis of variance (ANOVA) with Tukey’s and an adjusted *p* value (FDR) cutoff of 0.05 was used to identify statistically significant ions. Candidate metabolites were further positively identified through tandem mass spectrometry (MS/MS) and fragmentation patterns compared to pure chemicals and to spectra in the METLIN library. Partial least squares discriminant analysis (PLS-DA) scores plots of the first three components (prediction accuracy testing with 1000 permutations for validation of the model), heatmaps (Euclidean distance measure and Ward clustering algorithm), and enrichment analysis were also constructed through MetaboAnalyst 4.0. PLS-DA scores plots, heatmaps, and enrichment analysis were based on the congregate values of the metabolites validated through MS/MS. Visualization of the network interactions was performed through the Cytoscape plug-in Metscape [41].

Data were graphed with GraphPad Prism 6.0 as box and whisker plots (min to max). Longitudinal differences and inter-group assessment per month were analyzed with a two-way ANOVA with Tukey’s test for multiple correction testing. A *p* value of < 0.05 was considered statistically significant. Experimental description, tricarboxylic acid (TCA) cycle and purine metabolism pathways and graphs were constructed at BioRender.com (accessed on 25 January 2021).

### 2.4. Regression Analysis

Metabolites were grouped into the following 5 functional categories based on their roles in biochemical processes: (1) fatty acids: alpha-linolenic acid, arachidonic acid, docosahexaenoic acid, docosapentaenoic acid, eicosapentaenoic acid; (2) purine metabolism A: adenine, adenosine diphosphate (ADP), adenosine monophosphate (AMP), guanosine monophosphate (GMP), ribulose-5-phosphate; (3) purine metabolism B: guanine, hypoxanthine, inosine, uric acid, xanthine; (4) acylcarnitines: acetylcarnitine, carnitine, hexanoylcarnitine, *N6,N6,N6*-trimethyl-L-lysine, octanoylcarnitine, oleoylcarnitine; (5) energy metabolism: citric acid, fructose-6-phosphate, lactate, malate, succinate. To bring the error distribution closer to normal, we ln-transformed the signal intensities of all these metabolites, and calculated the means of ln-transformed signals for each of the 5 functional groupings. These data, along with the studied variables of interest (γ-ray dose, ^16^O ion dose, ^56^Fe ion dose, and time after irradiation), are provided in Supplementary Table S2. Visualization of correlations between variables in the data set was performed using *cor*, *cor.mtest* and *corrplot* in *R* (https://cran.r-project.org/web/packages/corrplot/index.html, accessed on 16 June 2020).

As an initial approach to analyze this data set, we considered multivariate linear regression with mean ln-transformed values of the metabolite groups as outcome variables and γ-ray dose, ^16^O and ^56^Fe ion doses, and time as predictors. However, diagnostics on regression residuals detected multiple influential outliers, so separate robust regressions on each outcome variable were used instead. Time^2^ terms and interactions between any of the radiation doses (γ, ^16^O or ^56^Fe ions) with time were considered, but were not used in the final analysis because of collinearity and/or lack of statistical significance.

The robust regression of mean ln-transformed signals for each metabolite group vs. γ-ray dose, ^16^O ion dose, ^56^Fe ion dose, and time was performed using the *lmrob* function from the *robustbase* package in *R* 4.0.2 software (https://www.rdocumentation.org/packages/robustbase/versions/0.93-6/topics/lmrob, accessed on 16 June 2020). Visualization of regression results was performed using the *visreg R* package (https://cran.r-project.org/web/packages/visreg/visreg.pdf, accessed on 24 June 2020).

## 3. Results

The weight of the mice in each time point did not differ significantly between groups (one-way ANOVA testing *p* < 0.05 for each month) (Supplementary Figure S1A). Candidate biomarkers from each time point that were selected for further evaluation were required to satisfy two parameters, an FDR corrected *p* value of <0.05 in at least one time point and a biologically relevant putative ID, as assigned through databases in Progenesis QI (NonLinear Dynamics, Inc., Newcastle, UK). Following MS/MS, 40 metabolites were positively identified, 23 in ESI^+^ and 17 in ESI^−^. Taurine and reduced glutathione were identified in both ionization modes. Twenty six metabolites were statistically significant at 1 month after exposure, 9 at 2 months, and 24 at 4 months (Supplementary Tables S3–S5). These Supplementary Tables also include mean values per group, standard errors of the mean (SEM) values, and log_2_ fold changes of each irradiated group compared to controls. At 1 month after exposure, responses showed an equal total number of increased or decreased metabolites comparatively to controls in all irradiation groups (Supplementary Table S3), whereas metabolites with >1.5 fold change (log_2_ of >0.58) were higher at the γ irradiated group (Supplementary Figure S1B). At 2 months after exposure, the balance of the overall responses remained similar within groups (Supplementary Table S4); however, the ^56^Fe exposed group had a higher number of metabolites showing decreased levels in relation to the other two exposures (Supplementary Figure S1B). However, at the 4 month time point, γ and ^56^Fe had similar responses, whereas ^16^O exhibited an increase in metabolites showing a fold change > 1.5 (log_2_ > 0.58) (Supplementary Table S5).

**Figure 2:**
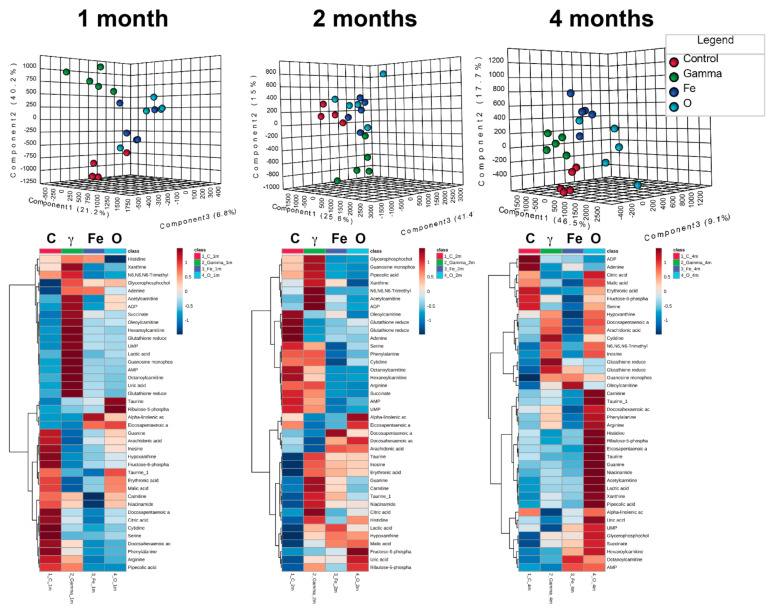
Multivariate data analysis on validated metabolites. Forty metabolites were identified through MS/MS and evaluated at 1, 2, and 4 months after irradiation. Distinct clustering in the partial least squares discriminant analysis (PLS-DA) scores plots is seen in all time points, while differential levels of the individual metabolites highlight the differences between the different exposures over time.

Multivariate data analysis utilizing the profiles of the 40 metabolites showed distinct clustering with 3D PLS-DA models. At months 1 and 2, the separation was primarily driven by the γ exposed group, while at month 4 the clustering was more defined between all four groups (Figure 2). At month 1, the percent of variation explaining the separation within groups was as follows: component 1 21.2%, component 2 40.2%, and component 3 6.8%, with an empirical *p* value for the permutation analysis of <0.001. At month 2, the percent of variation explaining the separation within groups was as follows: component 1 25.6%, component 2 15%, and component 3 41.4%, with an empirical *p* value for the permutation analysis of 0.032. At month 4, the % of variation explaining the separation within groups was as follows: component 1 46.5%, component 2 17.7%, and component 3 9.1%, with an empirical *p* value for the permutation analysis of 0.001. Hierarchical clustering heatmaps for each time point (Figure 2) further highlight the individual levels of each metabolite in each group. As can clearly be observed, exposure to γ had distinct responses from controls and HZE particles, while responses to the ^16^O exposure showed a delayed and amplified response (4 months).

**Figure 3:**
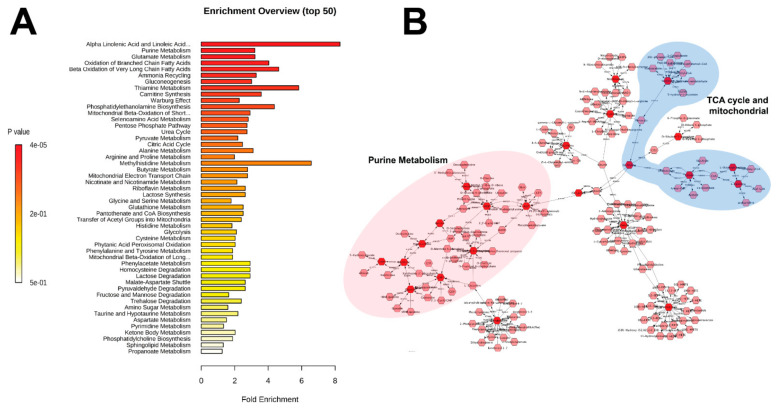
(**A**) Enrichment analysis of the MS/MS identified metabolites. Fatty acids and purine metabolism are prominently enriched. (**B**) Metscape analysis of metabolites (excluding fatty acids) demonstrates the connection between purine metabolism and tricarboxylic acid (TCA) cycle/mitochondrial metabolites. Dark red hexagons signify metabolites that were positively identified through MS/MS.

**Figure 4:**
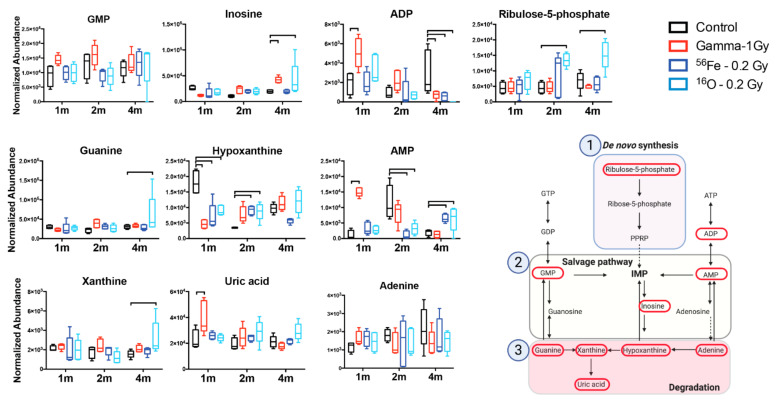
Specific changes in the purine metabolism highlight involvement of the whole pathway. Metabolic levels over 3 time points show the early responses to all exposure types, while the 4m time point shows persistent responses in the catabolic pathway specific to oxygen HZE irradiations. Brackets correspond to a *p* < 0.05 of an exposed group compared to control within that time point. Figure of the purine metabolism pathway was created with BioRender.com (accessed on 25 January 2021).

**Figure 5:**
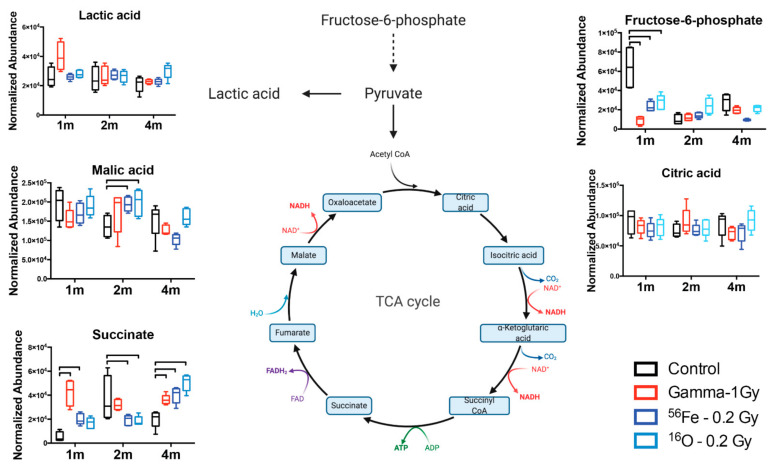
Changes in the TCA cycle highlight the dysregulation that increases with time and radiation quality. Brackets correspond to a *p* < 0.05 of an exposed group compared to control within that time point. TCA cycle figure was created with BioRender.com (accessed on 25 January 2021).

Pathway enrichment based on these metabolites showed fatty acid related processes (α-linolenic and linoleic acid metabolism, β-oxidation) in addition to purine metabolism and energy metabolism (TCA cycle, Warburg effect, gluconeogenesis) as a few of the most enriched pathways (Figure 3A). Metabolic pathway analysis through Metscape, with the exclusion of fatty acids (except arachidonic acid), displayed the connection between purine metabolism and mitochondrial/TCA cycle through pyruvate (not identified in this study) and lactate (Figure 3B), highlighting the delicate and interconnected balance between metabolic pathways in cells and tissues. Normalized levels of identified metabolites in these pathways are shown in Figure 4 and Figure 5 as box and whisker plots in a longitudinal manner. Early responses (1 month) in the purine metabolism showed significant effects primarily in the salvage and degradation pathways, more pronounced in the γ irradiated group (GMP, inosine, ADP, guanine, hypoxanthine, AMP, uric acid, adenine), and reduced responses in the HZE irradiated groups (inosine ADP, hypoxanthine, AMP, adenine). At 2 months after irradiation, persistent γ effects were concentrated primarily in the degradation pathway (guanine, hypoxanthine, xanthine), while HZE irradiations showed dampening of the responses with diffused pathway changes compared to controls (ribulose-5-phosphate, inosine, guanine, hypoxanthine, AMP). At the later time point (4 months), responses primarily in the degradation pathway were more prominent for the ^16^O exposure (ADP, inosine, guanine, AMP, xanthine, uric acid); however, it was evident that higher variation existed in the responses in that group (Figure 4).

Other prominent pathways or classes with important radiation induced-changes include the TCA cycle, acylcarnitines, fatty acids, and a small number of amino acids (Table 1, Supplementary Tables S3–S5). In the case of the mitochondria-related TCA cycle (Figure 5), early responses indicated reduced glycolysis with an increase in the Warburg effect for γ exposures, while the results of the TCA cycle intermediates indicated a disruption in the completion of the cycle in the conversion of succinate to fumarate for all radiation qualities. This occurred in a biphasic manner, with an increased production or accumulation at 1 month, the recovery of the metabolic process at 2 months, and finally leading to a significant increase in succinate in the spleen tissue compared to controls at 4 months after exposure. Changes in fatty acids, acylcarnitines, and amino acids were variable; however, a common pattern of increased levels at 4 months after exposure emerged for ^16^O exposures (Figure 2, Supplementary Table S5). Finally, increased levels of glycerophosphocholine and reduced glutathione at 1 and 4 months after irradiation revealed common radiation quality responses associated with membrane remodeling and oxidative stress, respectively.

**Figure 6:**
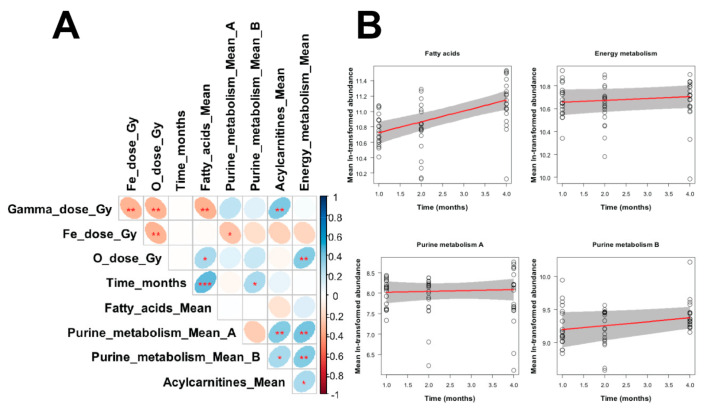
(**A**) Matrix of Pearson correlation coefficients between all variables in the analyzed data set. The meanings of all variables are provided in the main text, and a color-coded correlation scale is provided on the right of the plot. Blue ellipses represent positive correlations, and red ones represent negative correlations. Darker color tones and narrower ellipses represent larger correlation coefficient magnitudes. Red star symbols indicate statistical significance levels: *** indicates *p* < 0.001, ** indicates *p* < 0.01, * indicates *p* < 0.05, no stars indicates *p* > 0.05. These *p* values here are intended only for visualization: since the correlations are pairwise, without correction for multiple testing, only 3 star significance levels are likely to indicate strong associations. Blank squares indicate correlation coefficients close to zero. (**B**). Visualization of robust regression results for selected metabolite groupings vs. time after irradiation. Circles represent data points (mean ln-transformed signal intensities). Red lines represent regression fits, and grey shaded regions indicate estimated 95% confidence bands.

Regression results for the mean ln-transformed signals for each metabolite grouping vs. radiation doses (γ-rays, ^16^O or ^56^Fe ions) and time after irradiation are shown in Table 2 and Figure 6. Many of the regression parameters did not achieve statistical significance (Table 2), particularly considering that five regressions were performed. However, these results led to some useful conclusions about the patterns of these metabolite groups. Fatty acids were positively associated with time and negatively associated with γ-ray dose. Purine_metabolism_A (adenine, ADP, AMP, GMP, ribulose-5-phosphate) was positively associated with γ-ray dose. Purine_metabolism_B (guanine, hypoxanthine, inosine, uric acid, xanthine) was positively associated with time. Acylcarnitines had a marginally significant positive association with γ-ray dose. Energy metabolism was not significantly associated with any of the tested predictors.

## 4. Discussion

Future space missions will occur beyond LEO, with a return to the Moon including long-term boots on the ground objectives, and manned missions to Mars. Such long duration missions will expose astronauts to high cumulative doses of space radiation. It is estimated that a mission to Mars lasting between 650 and 920 days will lead to a dose equivalent of 870–1200 mSv (300–450 mGy) [42]. In this study, we investigated the responses of low dose HZE and γ ray whole body exposures on whole spleen tissue metabolism in a longitudinal manner with doses that would be accumulated during a long duration mission and defined responses in select pathways, such as energy and purine metabolism.

Several important metabolic pathways emerged that showed a biphasic response to radiation exposure and radiation quality. Purine metabolism is an important pathway for DNA and RNA synthesis, in addition to DNA repair and the generation of molecules that are critical components of nucleotides and cofactors [43]. Purine level maintenance is primarily through the de novo synthesis and salvage pathway, while the degradation pathway leads to the final step of uric acid production, which generally gets excreted in the urine. Uric acid itself has often been characterized as an anti-inflammatory molecule; however, new evidence suggests that it could be pro-inflammatory as increased levels can activate immune responses and lead to persistent inflammation [44,45]. Indeed, our studies identified perturbations in all three individual steps of the purine metabolism pathway. Early responses (1 month) were more prominent in the γ irradiated group and could have been driven by increased oxidative stress associated with the higher dose and nature of the sparsely ionizing radiation exposure generating high levels of free radicals through the hydrolysis of water. At 2 months after irradiation, overall responses were dampened, although HZE irradiations showed evidence of persistent perturbations. This may be reflective of increased DNA damage by the higher linear energy transfer (LET) particles (LET for ^16^O in this study is 14, LET for ^56^Fe in this study is ~170), previously characterized in spleen tissue by Chang et al. by measuring increased mutation frequency after exposure to ^56^Fe [46]. Indeed, persistent DNA damage was also reported in the blood of astronaut Scott Kelly in his one-year mission, who received an effective dose of 146.34 mSv, according to NASA reports [15]. However, at 4 months after irradiation, ^16^O exhibited persistent dysregulation in purine metabolism compared to the other exposures, spanning steps from the de novo synthesis to the degradation pathway. Dysregulation in this specific pathway in splenocytes after low dose x-ray irradiation was previously documented by Yamaoka et al. [47] and our findings are in agreement with their observations; however, we have extended our analysis in a longitudinal manner and identified novel increased responses of the splenic tissue to ^16^O exposure that could lead to long-term disability in effectively dealing with infections. Taken together, dysregulation in a key pathway of nucleotide pools that are necessary for cellular generation and effective DNA damage repair may play an important role in immune dysfunction, as defects in this pathway have been associated with severe immunodeficiency (e.g., adenosine deaminase) [48].

In terms of the bioenergetic capability of the spleen tissue following radiation exposure, significant imbalances were observed in the levels of specific intermediates of the TCA cycle, referred to collectively as energy metabolism in the regression analysis (Figure 6). The positive correlation with ^16^O suggested that a persistent overall perturbation of the pathway is present, which should be further evaluated. Early responses were more prominent in the γ-irradiated group, which could be due to a higher dose or the radiation quality. However, a prominent shift of persistent changes in the exposed groups, primarily in the HZE groups, was observed at 4 months after exposure, indicative also of mitochondrial dysregulation. Interestingly, succinate was significantly increased in those groups compared to the control, while malate, downstream from succinate, exhibited no significant changes. Fumarate was not identified in this study. This was not seen at 2 months where opposite patterns between succinate and malate suggested an effective completion of the pathway. The increased levels of succinate are of particular interest given the multidimensional role of this particular metabolite. While succinate plays an integral role in the TCA cycle, accumulation and rapid oxidation by succinate dehydrogenase can ultimately lead to a reverse electron transport and increased reactive oxygen species (ROS) [49]. This can be further augmented upon exposure to lipopolysaccharide (LPS) from bacteria, as seen in activated macrophages [50]. Alternatively, succinate can act as an immunometabolic signal by excretion from the cells and directing cell to cell communication and microenvironment responses to stress [51]. Finally, increased succinate levels can lead to epigenetic alterations and gene regulation [52,53], which can have detrimental effects in the activation of the immune system and responses to infections.

Taken together, the regression results mentioned above suggest that the strongest radiation responses were found for fatty acids and purine_metabolism_A. In both cases, ^16^O ions appeared to elicit stronger (although not statistically significant) responses per unit dose than γ-rays, whereas ^56^Fe ions tended to produce negative non-significant responses. These findings may reflect dependences of metabolite responses on factors such as radiation LET and the number of ionizing track traversals per cell at the tested doses. The interesting finding of positive fatty acid correlation with time and with ^16^O suggests the activation of pathways involving intermediates of inflammation. The fatty acids identified belong in the omega-6 and omega-3 categories, which are precursors for eicosanoid mediators such as prostaglandins, leukotrienes, and thromboxanes [54,55]. These intermediates can act as signaling molecules and control immune responses through regulating cytokine production from immune cells [54]. Changes in fatty acids following exposure to space radiation have been reported previously, not only in astronauts [15], but also in tissues from HZE exposed animals and spaceflight animals [56,57] and microgravity stimulated studies [58], including increased expression of enzymes such as COX2 [59]. Given also that common themes emerge for responses such as radiation or microgravity, it is imperative to investigate in future studies whether combined stressors (including CO2 and sleep deprivation among others) can lead to heightened responses and whether effective countermeasures can be designed to address some of the underlying metabolic changes.

Although the results from this study have provided significant insights into the long-term responses to radiation in the metabolic responses in the spleen, there are limitations that should be considered and addressed in future studies for any meaningful risk assessment. First, the space radiation environment is highly complex and astronauts will have a protracted exposure throughout their mission; therefore, immune related responses may be substantially different compared to acute exposures. Second, the immune composition of the spleen following radiation exposure was not assessed in this study, which could have indicated the main origin of these metabolic responses. Although secondary immune organs are impacted by radiation, it remains to be investigated if there is a persistent shift in cell populations within this tissue at a longer time point after the exposure and how this could impact the adaptive response. Some studies have shown that both B-cells and T-cells are impacted by radiation in the spleen [25,60], including in humanized mice [61]. However, long-term responses to ^28^Si irradiations did not reveal any differences in the population of humanized cells, compared to controls [61], or significant changes in the B- and T-cell populations in the spleen of mice [60] with ^56^Fe. However, Gridley et al. did conclude that the more complex space radiation environment may lead to a broader immune dysfunction [60]. Additional future studies should also incorporate data from multi-omic approaches, such as gene expression and proteomics data, to provide a systems biology overview of the tissue responses to space radiation.

As space radiation remains a significant risk for future missions and infections or the reactivation of viruses will need to be dealt with by the crew without a full scale medical facility, understanding the underlying responses will help design mitigators, pharmaceuticals, and even modified nutritional supplements that will be more effective. Combined exposures with microgravity analogs as an example may further elucidate any changes that may be due to a long duration deep space mission. Metabolomics, for example, as highlighted in this study, identified novel responses in the spleen in purine dysregulation, energy and fatty acid metabolism, which could potentially serve as substrates for countermeasure development. As common themes in responses emerge, such as in mitochondria dysfunction from animals to humans [56], it may be possible to monitor the health status of astronauts and provide corrective interventions [62].

## Figures and Tables

**Table 1:** Validate Metabolites through MS/MS.

Adduct	Metabolite	*m/z*	Ret. Time	Ppm Error	Two way ANOVA *p* Value (Time Factor)	Organic Compound Class
[M + H] +	Docosahexaenoic acid	329.2476	6.97	0.35	0.0003	Polyunsaturated fatty acid (PUFA)
[M + H] +	Phenylalanine	166.0866	0.49	2.21	0.0338	Amino acid
[M + H] +	Guanosine monophosphate (GMP)	364.0653	0.44	0.04	0.3411	Purine ribonucleoside monophosphate
[M + NH4] +	Xanthine	170.0658	0.33	9.33	0.4095	Purine derivative
[M + H] +	Oleoylcarnitine	426.3578	5.84	0.19	0.0107	Acylcarnitine
[M + H] +	Guanine	152.0571	0.44	3.03	0.0876	Purine derivative
[M + Na] +	Glycerophosphocholine	280.0925	0.38	2.23	<0.0001	Choline derivative
[M + H] +	Acetylcarnitine	204.1235	0.41	2.35	0.01	Acylcarnitine
[M + H] +	Octanoylcarnitine	288.2172	3.5	0.98	0.2444	Acylcarnitine
[M + H] +	Arginine	175.1194	0.4	2.54	0.1893	Amino acid
[M + H] +	Carnitine	162.1128	0.41	2.32	0.1449	Non-essential amino acid, vitamin
[M + H] +	*N6,N6,N6*-Trimethyl-L-lysine	189.1601	0.35	1.71	0.8555	Amino acid derivative
[M + H] +	Taurine	126.0223	0.45	3.26	0.0043	Amino acid derivative
[M − H] −	Taurine	124.0061	0.38	9.85	0.0438	
[M + H] +	Cytidine	244.0924	0.43	1.38	0.1413	Nucleoside
[M + H] +	Alpha-linolenic acid	279.2322	6.7	1.53	0.0114	PUFA
[M + Na] +	Inosine	291.0689	0.43	3.8	0.0007	Purine nucleoside
[M + Na] +	Hypoxanthine	159.0282	0.41	3.83	0.0048	Purine derivative
[M + Na] +	Adenosine monophosphate	370.0512	0.41	3.04	0.0448	Purine ribonucleoside monophosphate
[M + H] +	Hexanoylcarnitine	260.1854	2.69	0.77	0.1674	Acylcarnitine
[M + H] +	Nicotinamide (Niacinamide)	123.0555	0.43	1.69	0.0041	Pyridine derivative, vitamin
[M + H] +	Pipecolic acid	130.0865	0.35	2.64	0.3599	Amino acid derivative
[M + H] +	Docosapentaenoic acid	331.2647	7.2	4.77	<0.0001	PUFA
[M + H] +	Glutathione reduced	308.0912	0.43	0.64	0.0003	Amino acid derivative
[M − H] −	Glutathione reduced	306.0756	0.41	2.95	<0.0001	
[M − H] −	Citric acid	191.0184	0.41	6.81	0.8517	Weak organic acid
[M − H] −	Erythronic acid	135.0291	0.41	5.32	0.1656	Sugar acid
[M − H] −	Eicosapentaenoic acid	301.2162	6.63	3.49	0.1158	PUFA
[M − H] −	Histidine	154.0612	0.37	6.14	0.0419	Amino acid
[M − H] −	Lactic acid	89.0241	0.44	2.52	0.005	Organic acid
[M − H] −	Uric acid	167.02	0.41	6.12	0.0283	Purine derivative
[M − H] −	Adenosine diphosphate (ADP)	426.0229	0.45	1.8	<0.0001	Purine ribonucleoside diphosphate
[M − H] −	Fructose-6-phosphate	259.0217	0.43	2.57	<0.0001	Hexose phosphate
[M − H] −	Adenine	134.0459	0.38	10.3	0.5518	Purine base
[M − H] −	Succinate	117.0186	0.45	5.35	<0.0001	Dicarboxylic acid
[M − H] −	Serine	104.0347	0.37	0.88	0.0027	Amino acid
[M − H] −	Uridine monophosphate (UMP)	323.028	0.43	1.71	0.7291	Pyrimidine ribonucleoside monophosphate
[M − H] −	Ribulose-5-phosphate	229.0113	0.43	2.09	0.014	Pentose phosphate
[M − H] −	Malic acid	133.0133	0.41	6.79	0.0002	Dicarboxylic acid
[M − H] −	Arachidonic acid	303.2317	7.07	4.1	0.0605	PUFA

**Table 2:** Best-fit parameter values produced by robust linear regression for each metabolite grouping. For convenience, those parameter values for radiation or time response slopes that achieved *p* values < 0.05 are shown in bold font.

Metabolite Grouping	Robust Linear Regression Parameters			
	Meaning	Best-fit Value	Standard Error	*p* Value
Fatty acids	Intercept	10.585	0.095	<2 × 10^−16^
	Slope for gamma ray dose (Gy^−1^)	**−0.243**	0.106	0.025
	Slope for O ion dose (Gy^−1^)	0.88	0.572	0.13
	Slope for Fe ion dose (Gy^−1^)	−0.375	0.379	0.326
	Slope for time (months^−1^)	**0.141**	0.028	4.5 × 10^−6^
Purine metabolism A *	Intercept	7.997	0.185	<2 × 10^−16^
	Slope for gamma ray dose (Gy^−1^)	**0.392**	0.169	0.024
	Slope for O ion dose (Gy^−1^)	1.311	0.954	0.175
	Slope for Fe ion dose (Gy^−1^)	−0.147	0.679	0.829
	Slope for time (months^−1^)	0.023	0.062	0.712
Purine metabolism B #	Intercept	9.136	0.159	<2 × 10^−16^
	Slope for gamma ray dose (Gy^−1^)	0.136	0.115	0.241
	Slope for O ion dose (Gy^−1^)	0.771	0.666	0.253
	Slope for Fe ion dose (Gy^−1^)	−0.22	0.587	0.709
	Slope for time (months^−1^)	**0.06**	0.028	0.035
Acylcarnitines	Intercept	10.914	0.192	<2 × 10^−16^
	Slope for gamma ray dose (Gy^−1^)	0.319	0.162	0.054
	Slope for O ion dose (Gy^−1^)	0.154	0.95	0.872
	Slope for Fe ion dose (Gy^−1^)	−0.122	0.866	0.888
	Slope for time (months^−1^)	0.025	0.044	0.578
Energy metabolism	Intercept	10.639	0.066	<2 × 10^−16^
	Slope for gamma ray dose (Gy^−1^)	−0.017	0.063	0.784
	Slope for O ion dose (Gy^−1^)	0.502	0.296	0.096
	Slope for Fe ion dose (Gy^−1^)	−0.423	0.316	0.186
	Slope for time (months^−1^)	0.017	0.014	0.245

* adenine, ADP, AMP, GMP, ribulose-5-phosphate; # guanine, hypoxanthine, inosine, uric acid, xanthine.

## Data Availability

The raw chromatographic data are available through the GeneLab database [63] GLDS-360 https://doi.org/10.26030/wb0c-fm19 (accessed on 24 February 2021).

## Supplementary Materials

The following are available online at https://www.mdpi.com/1422-0067/22/6/3070/s1, Figure S1: (A) Weight of mice in grams (box and whisker plots), (B) The number of metabolites showing a fold change of >1.5 or <1.5 relative to controls at each time point, Table S1: Chromatographic and mass spectrometry conditions, Table S2: Data for regression analysis, Table S3: Month 1 results, Table S4: Month 2 results, Table S5: Month 4 results.
