# Supplementary material for: Effects of Low Dose Space Radiation Exposures on the Splenic Metabolome
Source: Int J Mol Sci. 2021 Mar 17;22(6):3070. doi: 10.3390/ijms22063070 (PMC8002539; doi:10.3390/ijms22063070)
Supplement: Supplementary file 1 [file ijms-22-03070-s001.pdf]

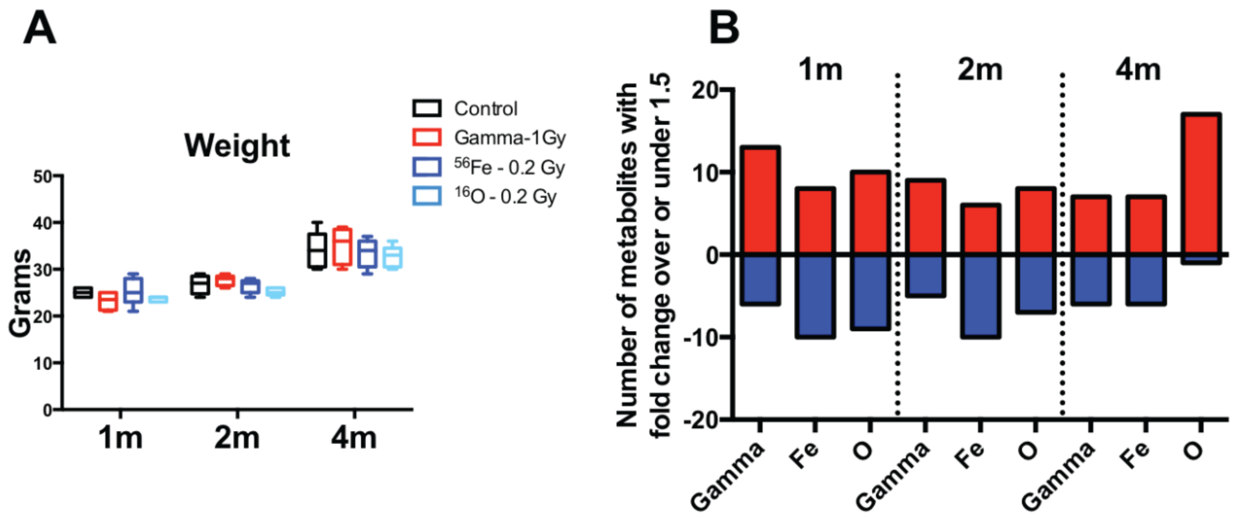

Supplementary Figure S1. Panel (**A**) Weight of mice in grams (box and whisker plots), Panel (**B**) The number of metabolites showing a fold change of >1.5 or <1.5 relative to controls at each time point.

Supplementary Table S1. Chromatographic and mass spectrometry conditions.

Chromatographic conditions for untargeted metabolomics

Step	Time (min)	Flow (mL/min)	% A	% B	%D	Curve Initial
1	Initial	0.4	97	3	0	6
2	0.5	0.4	97	3	0	6
3	4	0.4	40	60	0	6
4	8	0.4	2	98	0	6
5	9	0.4	0	5	95	6
6	10	0.4	0	5	95	6
7	11	0.4	25	25	50	6
8	13	0.4	97	3	0	6

Solvent A: Water+0.1% Formic acid

Solvent B: Acetonitrile+0.1% Formic acid

Solvent D: IPA/ACN 90/10+0.1%FA+10mM HCOONH4

Other parameters for tissue analysis:

**Instrument Parameter**	**UPLC TOFMS Xevo G2**	
Autosampler	Injection volume µL	2
Column Oven	Temperature °C	60
	Column	ACQUITY UPLC BEH C18 1.7µm
MS	Capillary Voltage kV	3
MSe function	Cone Voltage V	30
	Source Temp °C	120
	Desolvation Temp °C	500
	Cone Gas Flow (L/Hr)	25
	Desolvation Gas (L/Hr)	1000
	Collision Energy V	6

Supplementary Table S2. Data for regression analysis.

Gamma_dose_Gy	Fe_dose_Gy	O_dose_Gy	Time_months	Fatty_acids_Mean	Purine_metabolism_Mean_A	Purine_metabolism_Mean_B	Acylcarnitines_Mean	Energy_metabolism_Mean
0	0	0	1	10.8040468	8.128608418	9.562662415	11.43369065	10.83183084
0	0	0	1	10.7481201	7.579494031	9.44291115	10.54704029	10.33865232
0	0	0	1	10.8097759	7.596729949	9.614818974	11.11737712	10.67757462
0	0	0	1	10.9668432	7.62363612	9.674623038	11.0920235	10.59304813
1	0	0	1	10.355432	8.535555718	9.024640589	11.68652919	10.60303475
1	0	0	1	10.3738427	8.833018255	9.307780351	11.52807426	10.91635687
1	0	0	1	10.4749974	8.602450302	9.201540896	11.52195862	10.53708495
1	0	0	1	10.8249563	8.690100037	9.231952295	11.29115922	10.72150046
1	0	0	1	10.8281189	8.802899547	9.334996929	11.41427508	10.63055824
0	0.2	0	1	10.5863273	7.300667562	9.897969517	10.64282223	10.564966
0	0.2	0	1	10.5987557	8.022781962	8.791637826	10.03748289	10.56185937
0	0.2	0	1	10.814802	8.137578223	8.976491664	10.40967712	10.78674594
0	0.2	0	1	10.9767536	8.346595672	9.067092197	11.03901735	10.76025725
0	0.2	0	1	10.8179711	8.26979918	9.044496135	10.49314057	10.67677226
0	0	0.2	1	10.5867382	7.851671708	9.626676755	11.36674996	10.62269888
0	0	0.2	1	10.7459898	8.607617402	9.310967357	10.70451813	10.82529907
0	0	0.2	1	10.7166449	8.368210473	9.167671423	11.01318562	10.62321335
0	0	0.2	1	10.6994047	8.188580683	9.041445391	10.67428603	10.84029518
0	0	0.2	1	11.0641364	8.00220368	9.479024854	10.79885503	10.66328288
0	0	0	2	10.3453662	8.37500882	8.967809489	11.75138736	10.6408052
0	0	0	2	11.2658213	8.042493662	8.614389904	10.51233144	10.17994201
0	0	0	2	10.7822935	8.28033659	9.07762497	11.24742428	10.6845552
0	0	0	2	10.7663332	8.291339633	9.039678256	11.38313318	10.52668139
1	0	0	2	9.87952342	8.540332885	9.155860537	11.35436603	10.42855477
1	0	0	2	10.3072769	8.598087353	9.599208302	11.39289671	10.60545932
1	0	0	2	9.89282263	8.634604935	9.583343122	11.32435871	10.59655257
1	0	0	2	10.5076806	8.052110575	9.595240463	11.2597144	10.7718183

1	0	0	2	10.5204125	8.773303929	9.345954905	11.32310381	10.87931666
0	0.2	0	2	10.6125417	6.193109417	9.369168235	10.91895019	10.48020492
0	0.2	0	2	10.7711549	6.776226112	9.517329658	11.00306224	10.50951805
0	0.2	0	2	10.7655321	8.152977271	9.161538487	10.67727669	10.78621382
0	0.2	0	2	11.0066724	7.971310262	9.424066076	11.12946935	10.61534419
0	0.2	0	2	10.9184113	8.149240016	9.452828624	11.18778671	10.60574146
0	0	0.2	2	10.4381425	8.12272208	9.484963967	10.75042568	10.81130305
0	0	0.2	2	11.2008312	7.829240108	8.734871682	10.50198913	10.44330126
0	0	0.2	2	11.344846	7.920124271	9.607559676	11.28792395	10.90045636
0	0	0.2	2	11.4647614	7.837612657	9.141685971	10.70973678	10.55345142
0	0	0.2	2	11.0096453	8.158543483	9.283304934	10.56441553	10.76125549
0	0	0	4	10.9736129	7.570673765	9.237763286	10.24679705	9.984723647
0	0	0	4	11.1468909	8.632823486	9.228770287	10.96230883	10.71177432
0	0	0	4	11.2254005	8.202961651	9.441304909	10.87928503	10.8360947
0	0	0	4	11.0978708	8.213058771	9.267442472	10.53675742	10.79071204
0	0	0	4	11.0972787	8.072195994	9.449968446	10.95798283	10.83233124
1	0	0	4	10.9691189	8.029222335	9.573184274	11.01665386	10.70045199
1	0	0	4	11.257311	6.9110316	9.492110484	11.07438138	10.55588536
1	0	0	4	11.2903529	7.706396063	9.492092364	11.029805	10.56242314
1	0	0	4	10.7882953	8.057877872	9.466936974	10.99197526	10.70087069
1	0	0	4	11.1755684	7.985821509	9.745874942	11.15360529	10.77409646
0	0.2	0	4	10.6940488	8.422263545	9.311023776	11.28459118	10.52833755
0	0.2	0	4	10.7968586	8.100787805	9.412487405	11.25230157	10.59802712
0	0.2	0	4	10.9718824	8.171376775	9.241224416	10.89917672	10.53692939
0	0.2	0	4	11.2556145	7.702745564	9.177933559	11.24564699	10.53548203
0	0.2	0	4	10.9547265	7.637181404	9.110590705	11.31376951	10.24467109
0	0	0.2	4	10.3011824	6.371699373	10.37214894	11.00494429	10.77646163
0	0	0.2	4	11.6930345	8.975685568	9.475438974	11.52582287	10.85392059
0	0	0.2	4	11.4362347	9.028515028	9.727041486	11.6174915	10.93599983
0	0	0.2	4	11.676015	8.895628029	9.804430041	11.37202741	10.99913881
0	0	0.2	4	10.9919912	8.86421488	9.468087601	11.28597432	10.91431742

Supplementary Table S3.
Month 1 results.

Metabolite	FDR corrected ANOVA p-value	Control - 1m		Gamma - 1m			Fe - 1m			O - 1m		
		Mean	SEM	Mean	SEM	Log2 Fold Change	Mean	SEM	Log2 Fold Change	Mean	SEM	Log2 Fold Change
Docosahexaenoic acid	0.0011626	107234.2	3532.5	78596.6	8982.9	-0.45	54885.2	6084.3	-0.97	60750.9	5061.9	-0.82
Phenylalanine	0.012339	118295.8	9912.3	83577.1	4359.8	-0.50	64510.5	12524.3	-0.87	68742.0	8415.3	-0.78
Guanosine monophosphate	0.048582	9092.8	1845.0	14404.8	743.1	0.66	9865.3	1026.3	0.12	9918.0	1358.3	0.13
Xanthine	*NS*	2108.7	150.6	2341.0	141.2	0.15	1929.6	643.6	-0.13	1986.9	483.2	-0.09
Oleoylcarnitine	0.0012621	377844.3	70942.7	1044120.7	75186.2	1.47	545627.6	88931.0	0.53	583303.3	90854.5	0.63
Guanine	*NS*	29901.6	2169.2	22376.2	1625.5	-0.42	24947.9	7212.8	-0.26	26780.9	2547.0	-0.16
Glycerophosphocholine	0.0046895	9693.0	1561.1	31513.9	2809.0	1.70	24602.2	3765.0	1.34	26367.3	3338.0	1.44
Acetylcarnitine	0.042288	366093.7	9587.5	499698.2	25409.0	0.45	349477.7	40549.2	-0.07	420233.8	41137.9	0.20
Octanoylcarnitine	5.35E-06	145.9	145.9	13378.5	1588.9	6.52	2019.7	875.7	3.79	1148.5	541.2	2.98

Arginine	0.0046895	72803.4	7257.5	59731.7	2734.6	-0.29	35751.6	6415.5	-1.03	41928.0	6085.5	-0.80
Carnitine	*NS*	555338.9	42877.4	526811.1	37508.3	-0.08	411212.6	59401.4	-0.43	527429.9	57212.9	-0.07
N6,N6,N6-Trimethyl-L-lysine	0.00093904	4772.4	800.7	5727.1	527.3	0.26	1997.0	336.2	-1.26	2443.7	396.8	-0.97
Taurine (pos mode)	0.025201	367493.6	24779.2	320911.7	29445.7	-0.20	306224.2	49418.7	-0.26	371654.8	34894.3	0.02
Cytidine	0.0050888	2067.9	319.8	702.2	311.3	-1.56	400.7	178.0	-2.37	591.5	208.5	-1.81
Alpha-linolenic acid	0.048102	8321.7	2346.0	8179.3	2862.7	-0.02	21057.7	1867.2	1.34	14886.1	4441.8	0.84
Inosine	*NS*	26147.8	2008.6	12042.3	770.4	-1.12	15519.7	5165.1	-0.75	18495.8	2429.0	-0.50
Hypoxanthine	0.00028513	18053.2	1947.2	4647.3	600.3	-1.96	7097.9	1871.2	-1.35	9014.4	698.7	-1.00
AMP	7.49E-08	1024.7	773.0	14949.9	648.4	3.87	3254.5	881.4	1.67	2826.3	527.0	1.46
Hexanoylcarnitine	0.00093904	8959.2	1801.0	46153.7	7222.3	2.37	17186.2	3896.8	0.94	15343.3	2734.6	0.78
Niacinamide	*NS*	152988.1	7882.4	144826.8	3366.0	-0.08	125387.4	15123.8	-0.29	145906.5	12774.0	-0.07
Pipecolic acid	0.016862	22160.8	1719.8	20664.7	1328.5	-0.10	14037.0	2398.2	-0.66	13939.7	1528.4	-0.67
Docosapentaenoic acid	0.0017399	55399.9	3998.0	35802.4	2055.1	-0.63	27508.3	3463.3	-1.01	35637.4	4078.3	-0.64
Glutathione reduced (pos mode)	0.0025272	203942.3	7270.3	425981.6	19865.8	1.06	258949.7	46547.6	0.34	255271.8	28688.4	0.32
Citric acid	*NS*	91779.3	10454.3	81808.4	5574.9	-0.17	75696.4	5961.2	-0.28	81232.7	6763.9	-0.18

Erythronic acid	0.0040865	82235.3	9751.9	41881.6	3969.6	-0.97	56170.0	4820.0	-0.55	76778.4	6734.1	-0.10
Eicosapentaenoic acid	*NS*	24753.7	1550.6	23541.0	1968.2	-0.07	34052.4	6251.8	0.46	35090.5	5632.9	0.50
Histidine	*NS*	10382.5	1157.2	10820.4	430.0	0.06	10715.4	2321.9	0.05	9057.5	740.3	-0.20
Lactic acid	0.012339	25743.1	3442.2	40128.3	4286.7	0.64	25976.3	904.8	0.01	27991.0	1115.1	0.12
Uric acid	0.022237	22408.6	3945.6	39692.2	5647.9	0.82	25825.1	1176.4	0.20	24217.3	1161.2	0.11
Taurine (neg mode)	*NS*	1003060.7	90297.0	1096304.8	53741.2	0.13	1125574.4	71676.8	0.17	1348918.1	50086.1	0.43
ADP	0.016853	1975.5	588.3	5055.5	692.0	1.36	1980.4	504.0	0.00	3319.7	641.1	0.75
Fructose-6-phosphate	0.00025866	64033.7	11610.6	8996.3	1931.8	-2.83	23603.2	2297.3	-1.44	27976.2	3454.1	-1.19
Adenine	*NS*	1171.8	139.2	1643.2	168.2	0.49	1636.7	196.3	0.48	1436.4	234.3	0.29
Succinate	7.07E-05	5148.2	2093.2	41983.6	4781.4	3.03	19984.8	2065.7	1.96	16777.6	2045.7	1.70
Serine	*NS*	1585.6	227.8	1069.7	153.6	-0.57	1075.2	76.6	-0.56	1132.0	149.9	-0.49
UMP	0.00013466	3956.5	1196.2	24446.4	3088.8	2.63	9547.9	1190.3	1.27	8703.5	1280.1	1.14
Ribulose-5-phosphate	*NS*	4502.3	909.4	4778.5	823.6	0.09	4971.5	1254.5	0.14	6953.7	1345.7	0.63
Malic acid	*NS*	195108.8	21636.9	154891.9	11853.9	-0.33	169975.1	11909.3	-0.20	189271.6	12715.0	-0.04
Arachidonic acid	*NS*	306753.6	22687.5	227611.2	15061.3	-0.43	263115.0	24962.3	-0.22	271088.5	16160.7	-0.18
Glutathione reduced (neg mode)	0.000222	95186.7	11556.1	353154.5	20636.5	1.89	145182.8	38856.8	0.61	109685.3	28905.5	0.20

Supplementary Table S4.
Month 2 results.

		Control - 1m		Gamma - 1m			Fe - 1m			O - 1m		
**Metabolite**	**FDR corrected ANOVA p-value**	**Mean**	**SEM**	**Mean**	**SEM**	**Log2 Fold Change**	**Mean**	**SEM**	**Log2 Fold Change**	**Mean**	**SEM**	**Log2 Fold Change**
Docosahexaenoic acid	*NS*	57017.2	5330.7	46148.5	7740.4	-0.31	65398.4	6846.5	0.20	72170.2	8497.3	0.34
Phenylalanine	*NS*	74129.8	12563.0	74241.6	8383.5	0.00	64952.9	7947.2	-0.19	67478.0	11176.2	-0.14
Guanosine monophosphate	*NS*	12711.4	2293.1	16082.2	1684.2	0.34	9268.4	1060.5	-0.46	8768.6	1527.6	-0.54
Xanthine	*NS*	1790.1	322.1	2412.0	300.5	0.43	1908.3	246.8	0.09	1213.7	278.2	-0.56
Oleoylcarnitine	*NS*	880474.2	187243.3	635084.0	61809.5	-0.47	696001.8	129017.9	-0.34	561178.5	80031.6	-0.65
Guanine	*NS*	21790.5	3118.8	38735.9	4734.1	0.83	30250.4	2640.7	0.47	26524.8	3980.4	0.28
Glycerophosphocholine	*NS*	35943.1	5562.7	40520.8	3130.2	0.17	31019.4	3562.0	-0.21	31440.1	2064.6	-0.19
Acetylcarnitine	*NS*	444579.4	66593.2	619195.5	43615.0	0.48	483581.8	48128.3	0.12	427951.7	57221.8	-0.05
Octanoylcarnitine	*NS*	10953.3	4141.8	7225.8	1025.4	-0.60	2828.9	928.0	-1.95	2909.3	1096.0	-1.91
Arginine	*NS*	51613.9	14881.1	52396.9	3315.3	0.02	38449.5	3930.6	-0.42	37708.6	6882.5	-0.45
Carnitine	*NS*	460510.3	61692.9	663427.0	56530.7	0.53	563573.4	59474.0	0.29	502590.5	66467.6	0.13

N6,N6,N6-Trimethyl-L-lysine	0.034954	3643.6	864.0	6207.1	838.0	0.77	2821.3	425.5	-0.37	2840.7	473.1	-0.36
Taurine (pos mode)	0.047004	279999.8	40694.7	546149.4	38444.0	0.96	463462.5	54990.9	0.73	390713.3	50002.9	0.48
Cytidine	*NS*	678.4	259.6	692.4	209.0	0.03	368.7	256.4	-0.88	527.6	421.5	-0.36
Alpha-linolenic acid	*NS*	29235.8	15451.4	10603.5	4365.5	-1.46	16067.7	1787.9	-0.86	50064.4	20737.7	0.78
Inosine	0.047004	10669.6	1097.2	23209.4	3180.3	1.12	20455.9	1219.6	0.94	19845.9	2304.9	0.90
Hypoxanthine	0.034954	3451.6	84.5	7715.2	1203.7	1.16	9067.3	616.7	1.39	8695.3	1247.1	1.33
AMP	0.018483	11329.4	2854.1	8327.9	1619.9	-0.44	1166.5	565.9	-3.28	3102.9	848.4	-1.87
Hexanoylcarnitine	*NS*	49080.4	17361.5	33072.8	3472.7	-0.57	17241.2	2294.1	-1.51	18579.6	3495.3	-1.40
Niacinamide	*NS*	130131.7	15502.3	185345.3	15701.7	0.51	160267.8	18713.3	0.30	159570.5	25398.2	0.29
Pipecolic acid	*NS*	17673.4	3741.7	20178.3	607.7	0.19	14099.4	1094.3	-0.33	13441.3	1812.2	-0.39
Docosapentaenoic acid	*NS*	29721.6	4233.6	27896.1	3801.9	-0.09	37569.7	5206.1	0.34	32337.8	5427.6	0.12
Glutathione reduced (pos mode)	*NS*	350969.0	78587.5	260478.7	67222.0	-0.43	275295.1	34504.9	-0.35	279948.3	38067.5	-0.33
Citric acid	*NS*	74318.7	5642.2	90007.2	9930.8	0.28	75753.5	4435.8	0.03	79744.6	6437.7	0.10
Erythronic acid	*NS*	32406.9	2649.0	66248.1	18416.4	1.03	60545.4	7272.9	0.90	57413.7	7141.0	0.83
Eicosapentaenoic acid	*NS*	66574.3	38254.8	17117.7	3164.0	-1.96	30518.4	5018.1	-1.13	78187.3	24568.8	0.23
Histidine	*NS*	7455.0	1231.1	9866.7	985.3	0.40	8298.5	209.2	0.15	9405.3	1169.1	0.34

Lactic acid	*NS*	24494.8	4281.2	26659.9	2865.2	0.12	27337.2	1330.5	0.16	26267.5	1785.7	0.10
Uric acid	*NS*	19283.1	2388.2	24646.0	3558.3	0.35	24301.1	1027.8	0.33	28991.6	4144.1	0.59
Taurine (neg mode)	0.034954	915035.4	101592.7	1519379.9	96903.3	0.73	1322174.2	28294.0	0.53	1334734.1	126689.1	0.54
ADP	*NS*	885.0	286.2	2160.0	466.4	1.29	921.6	650.8	0.06	679.7	157.3	-0.38
Fructose-6-phosphate	0.034954	9604.7	2661.3	11878.5	1507.1	0.31	14202.3	1324.3	0.56	24074.8	3695.2	1.33
Adenine	*NS*	1800.6	168.4	1322.2	273.0	-0.45	1408.4	567.9	-0.35	1379.4	326.7	-0.38
Succinate	*NS*	36225.0	9525.7	32073.2	1918.1	-0.18	19177.7	1888.8	-0.92	18427.0	1759.6	-0.98
Serine	*NS*	1105.4	157.1	966.8	182.7	-0.19	576.6	232.0	-0.94	787.1	126.0	-0.49
UMP	0.018483	18669.7	3141.2	15371.0	2520.1	-0.28	4745.0	914.3	-1.98	6654.9	1411.7	-1.49
Ribulose-5-phosphate	*NS*	5786.0	1753.5	11940.7	1624.7	1.05	8735.4	3040.5	0.59	13520.6	953.6	1.22
Malic acid	*NS*	136466.6	14156.0	172923.6	24079.7	0.34	194287.8	8374.7	0.51	198400.9	15329.4	0.54
Arachidonic acid	0.018483	189095.7	25128.8	114019.4	14514.8	-0.73	302586.2	27883.9	0.68	292959.5	42535.1	0.63
Glutathione reduced (neg mode)	*NS*	224876.4	73221.5	59342.4	23825.5	-1.92	85187.8	25380.9	-1.40	114015.9	35034.3	-0.98

Supplementary Table S5.
Month 4 results.

Metabolite	FDR corrected ANOVA p-value	Control - 4m		Gamma - 4m			Fe - 4m			O - 4m		
		Mean	SEM	Mean	SEM	Log2 Fold Change	Mean	SEM	Log2 Fold Change	Mean	SEM	Log2 Fold Change
Docosahexaenoic acid	NS	83220.5211	5282.36616	87843.4753	5626.61268	0.08	65988.7941	3461.67659	-0.33	102403.619	16613.8353	0.3
Phenylalanine	0.020497	77998.2084	10233.5909	91603.9984	5809.72602	0.23	66590.7412	6000.57596	-0.23	110136.978	9624.95615	0.5
Guanosine monophosphate	NS	11400.1295	1317.02495	13160.6505	1543.44487	0.21	13211.3725	2128.33887	0.21	12845.8841	3244.99861	0.17
Xanthine	NS	1551.48631	174.986997	2105.26476	161.034915	0.44	1884.08975	179.515629	0.28	3226.16506	788.888735	1.06
Oleoylcarnitine	NS	676918.137	96541.5121	842719.058	42518.119	0.32	1022072.53	64999.3669	0.59	815363.145	94841.3436	0.27
Guanine	NS	30420.6108	2127.04118	32591.658	1912.99443	0.1	28605.3843	2950.92425	-0.09	60722.5877	23446.7012	1
Glycerophosphocholine	0.0086623	30462.8519	670.977912	43548.9778	3899.40123	0.52	43315.0723	2272.96441	0.51	54749.8832	5720.27039	0.85
Acetylcarnitine	0.0086623	412302.475	40777.9184	457741.106	23886.1341	0.15	451325.112	25411.3587	0.13	633304.537	48562.6236	0.62
Octanoylcarnitine	0.029983	2172.37894	518.646201	3471.47913	227.942689	0.68	8948.83036	2086.0717	2.04	7359.51703	2092.69279	1.76
Arginine	0.0086623	45364.348	4555.64632	52015.7616	1850.00692	0.2	41069.8555	915.888789	-0.14	62284.7285	4637.87633	0.46

Carnitine	0.016436	525494.083	50427.2669	567367.895	28012.8982	0.11	480750.582	31274.0114	-0.13	753574.985	73999.0741	0.52
N6,N6,N6-Trimethyl-L-lysine	0.016436	2540.37322	401.108182	5160.57173	747.363832	1.02	3099.27589	171.973754	0.29	5051.62864	723.992599	0.99
Taurine (pos mode)	0.016436	402441.089	35764.0666	415838.628	15599.525	0.05	364414.372	30997.7974	-0.14	543415.456	42114.2156	0.43
Cytidine	0.0057163	382.549522	178.119845	1597.2951	281.185363	2.06	98.6925345	94.4683557	-1.95	834.954773	253.549313	1.13
Alpha-linolenic acid	NS	40054.9242	10486.6706	22694.4163	4524.04398	-0.82	33266.2214	9802.28445	-0.27	40865.6152	12943.7613	0.03
Inosine	NS	19747.5252	1358.21958	42061.5211	2732.18636	1.09	19498.0789	1399.74052	-0.02	42604.1997	14823.2341	1.11
Hypoxanthine	0.011585	9678.60267	683.238408	11370.6042	1044.4528	0.23	5726.59494	371.975248	-0.76	11961.0331	1731.66338	0.31
AMP	0.0074368	1913.29695	422.718358	1306.4414	461.976836	-0.55	6552.48477	575.169661	1.78	6315.52461	1747.74128	1.72
Hexanoylcarnitine	0.0043865	14292.6193	2102.74628	17840.8582	2176.31746	0.32	30200.4579	4836.77684	1.08	38585.516	4118.77203	1.43
Niacinamide	0.011585	157301.033	16964.6111	170265.251	9122.78016	0.11	158598.682	8719.76129	0.01	236910.847	20472.665	0.59
Pipecolic acid	0.0070926	15157.4799	1192.84045	17333.5191	1079.86406	0.19	15723.9677	760.941364	0.05	23697.963	1995.11106	0.64
Docosapentaenoic acid	NS	49979.5677	6865.30757	59939.5763	3537.27676	0.26	42788.3172	1486.15803	-0.22	61547.3356	12407.9195	0.3
Glutathione reduced (pos mode)	0.00031387	28913.3445	13804.2319	392387.696	21319.37	3.76	156617.346	30896.1051	2.44	134565.349	56066.2639	2.22
Citric acid	NS	85574.2691	9381.84345	70803.3673	4492.27005	-0.27	72372.1362	7382.88669	-0.24	92208.0771	7999.25407	0.11

Erythronic acid	NS	84287.0549	10903.7487	53782.0213	4227.18288	-0.65	55424.2037	8234.15267	-0.6	73591.7828	16052.8436	-0.2
Eicosapentaenoic acid	NS	42444.1633	5173.53217	41120.5949	3152.24115	-0.05	37357.9509	4559.01458	-0.18	63227.6546	19642.6086	0.57
Histidine	0.0086623	8082.07461	1062.6001	6988.03332	417.171455	-0.21	7591.11488	449.47105	-0.09	11115.4604	492.376137	0.46
Lactic acid	0.021215	21087.3988	2431.98058	22725.6081	658.115526	0.11	22718.7484	953.399188	0.11	30127.4774	2344.47467	0.51
Uric acid	0.016436	21226.7752	2016.76096	17854.9249	1068.19287	-0.25	21744.884	854.011824	0.03	28923.2099	3023.86297	0.45
Taurine (neg mode)	NS	1258905.05	166617.515	1174846.53	51228.5323	-0.1	1169613.62	112105.529	-0.11	1605319.39	53638.1539	0.35
ADP	0.013639	2961.90496	1008.32821	731.146328	188.403409	-2.02	550.082347	185.477138	-2.43	0	0	-
Fructose-6-phosphate	0.0027938	28073.0115	4024.04919	19509.7942	1457.51756	-0.52	9327.01321	454.783463	-1.59	21446.8734	1524.15571	-0.39
Adenine	NS	2224.02317	505.042813	1403.2908	299.622802	-0.66	1693.34728	453.19723	-0.39	1480.43439	252.176793	-0.59
Succinate	0.00031387	20186.7123	3293.14986	36207.4226	1832.53938	0.84	40001.2153	3063.78936	0.99	50547.1065	3113.59904	1.32
Serine	NS	1078.75046	158.690403	722.384179	200.059461	-0.58	567.971158	119.151362	-0.93	929.781925	153.873792	-0.21
UMP	NS	10157.3694	1519.13036	5939.91469	814.633188	-0.77	11612.2773	634.439067	0.19	14448.8454	3978.98061	0.51
Ribulose-5-phosphate	0.0026523	6833.00263	1382.58997	4784.91374	231.27536	-0.51	5845.83444	973.600947	-0.23	15122.6203	2083.71996	1.15
Malic acid	0.029028	152876.851	20662.0645	126612.129	5667.62212	-0.27	103052.784	7001.55282	-0.57	160608.678	9518.04724	0.07
Arachidonic acid	NS	240508.014	27756.8279	295576.775	21965.7194	0.3	197969.044	8503.4489	-0.28	292123.44	48978.0738	0.28

Glutathione reduced (neg mode)	0.022675	8774.6252	6291.74383	99966.6296	12326.3626	3.51	66982.1543	18026.5653	2.93	43707.6598	25842.4085	2.32
